# Tris(1,10-phenanthroline-5,6-dione-κ^2^ *N*,*N*′)zinc bis­(perchlorate) acetonitrile monosolvate

**DOI:** 10.1107/S1600536811037081

**Published:** 2011-09-30

**Authors:** Jing Zhao, Heng Zhang, Zhaozhi Zhang, Haiyan Zhao, Guoyi Zhu

**Affiliations:** a Changchun Institute of Applied Chemistry, Chinese Academy of Sciences, Changchun 130022, People’s Republic of China; b Instrumental Analysis Center, Hebei Normal University, Shijiazhuang 050016, People’s Republic of China; c Department of Applied Chemistry, Hengshui University, Hengshui 053000, People’s Republic of China; d College of Science, Hebei University of Science and Technology, Shijiazhuang 050018, People’s Republic of China

## Abstract

In the title compound, [Zn(C_12_H_6_N_2_O_2_)_3_](ClO_4_)_2_·CH_3_CN, the Zn^II^ atom is coordinated by six N atoms from three chelating 1,10-phenanthroline-5,6-dione ligands in a distorted octa­hedral environment. In the crystal, inter­molecular C—H⋯O hydrogen bonds and O⋯π and N⋯π inter­actions [O⋯centroid distances = 2.907 (5) and 2.843 (7) Å; N⋯centroid distance = 2.861 (10) Å] link the complex cations, perchlorate anions and acetonitrile solvent mol­ecules into a three-dimensional network.

## Related literature

For the coordination chemistry of 1,10-phenanthroline-5,6-dione, see: Brechin *et al.* (2008 ▶); Khalaji *et al.* (2007 ▶); Ma *et al.* (2010 ▶); Rezvani *et al.* (2010 ▶). For the biological and electrochemical properties of transition metal complexes with 1,10-phenanthroline-5,6-dione, see: Boghaei & Asl (2007 ▶); Goss & Abruna (1985 ▶); Kou *et al.* (2009 ▶).
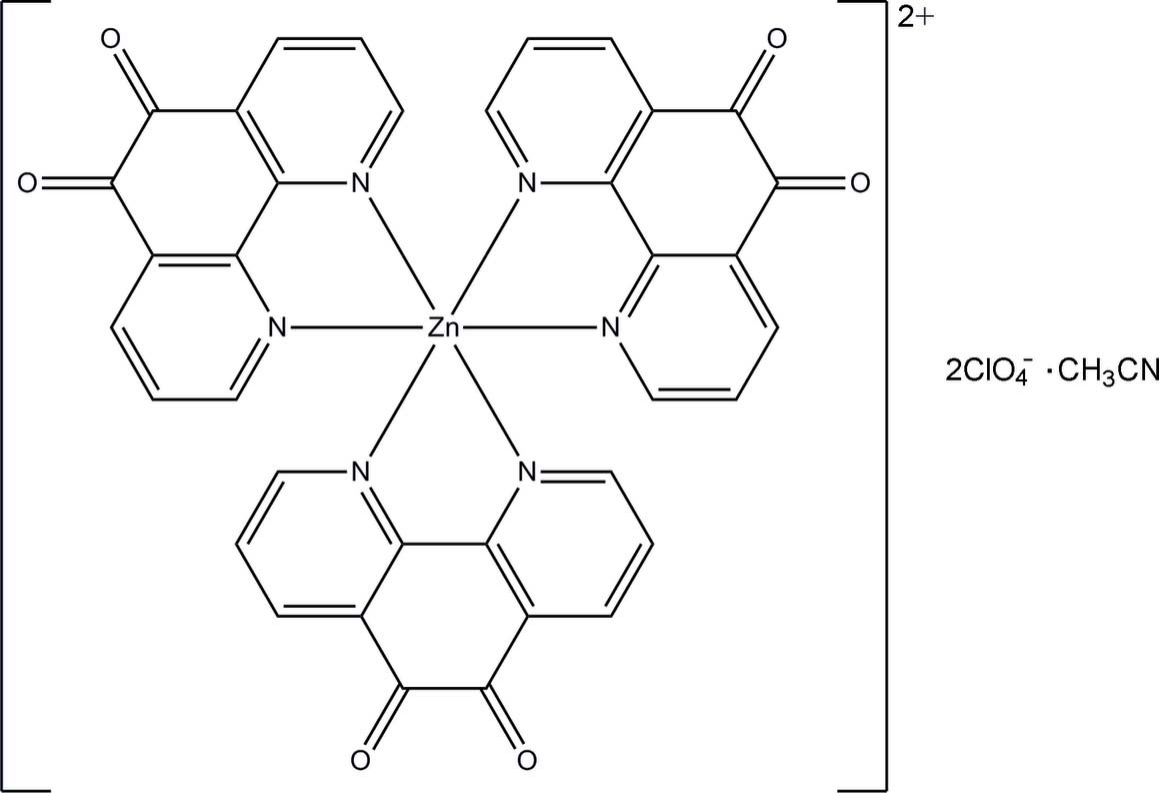


## Experimental

### 

#### Crystal data

[Zn(C_12_H_6_N_2_O_2_)_3_](ClO_4_)_2_·C_2_H_3_N
*M* *_r_* = 935.91
Orthorhombic, 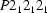

*a* = 13.446 (6) Å
*b* = 14.125 (6) Å
*c* = 20.483 (8) Å
*V* = 3890 (3) Å^3^
*Z* = 4
Mo *K*α radiation
μ = 0.85 mm^−1^
*T* = 296 K
0.30 × 0.12 × 0.12 mm

#### Data collection

Bruker APEXII CCD diffractometer
Absorption correction: multi-scan (*SADABS*; Sheldrick, 1996 ▶) *T*_min_ = 0.785, *T*_max_ = 0.905
23200 measured reflections
9569 independent reflections
5533 reflections with *I* > 2σ(*I*)
*R*_int_ = 0.079

#### Refinement

*R*[*F* ^2^ > 2σ(*F* ^2^)] = 0.075
*wR*(*F* ^2^) = 0.220
*S* = 0.97
9569 reflections
562 parameters
1 restraint
H-atom parameters constrained
Δρ_max_ = 1.16 e Å^−3^
Δρ_min_ = −0.39 e Å^−3^
Absolute structure: Flack (1983 ▶), 4095 Friedel pairs
Flack parameter: 0.12 (2)

### 

Data collection: *APEX2* (Bruker, 2007 ▶); cell refinement: *SAINT* (Bruker, 2007 ▶); data reduction: *SAINT*; program(s) used to solve structure: *SHELXS97* (Sheldrick, 2008 ▶); program(s) used to refine structure: *SHELXL97* (Sheldrick, 2008 ▶); molecular graphics: *SHELXTL* (Sheldrick, 2008 ▶); software used to prepare material for publication: *SHELXTL*.

## Supplementary Material

Crystal structure: contains datablock(s) I, global. DOI: 10.1107/S1600536811037081/hy2471sup1.cif

Structure factors: contains datablock(s) I. DOI: 10.1107/S1600536811037081/hy2471Isup2.hkl

Additional supplementary materials:  crystallographic information; 3D view; checkCIF report

## Figures and Tables

**Table 1:** Hydrogen-bond geometry (Å, °)

*D*—H⋯*A*	*D*—H	H⋯*A*	*D*⋯*A*	*D*—H⋯*A*
C1—H14⋯O13^i^	0.93	2.51	3.238 (9)	135
C2—H15⋯O3^ii^	0.93	2.58	3.030 (13)	110
C10—H12⋯O7^iii^	0.93	2.59	3.513 (14)	170
C25—H17⋯O4^ii^	0.93	2.51	3.136 (15)	125
C24—H26⋯O2^iv^	0.93	2.36	3.036 (10)	129
C34—H7⋯O9^v^	0.93	2.47	3.302 (12)	150
C38—H38*A*⋯O3^vi^	0.96	2.51	2.995 (16)	112
C38—H38*B*⋯O13^vii^	0.96	2.51	3.436 (19)	163

Symmetry codes: (i) 
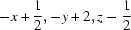
; (ii) 
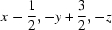
; (iii) 
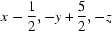
; (iv) 
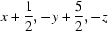
; (v) 
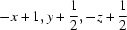
; (vi) 
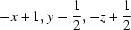
; (vii) 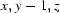.

**Fig. 1.**
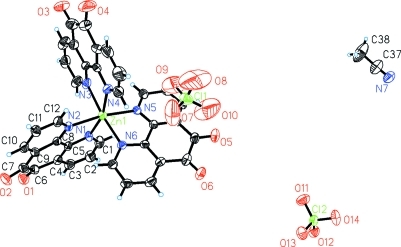
Molecular structure of the title compound. Displacement ellipsoids are drawn at the 30% probability level.

**Fig. 2.**
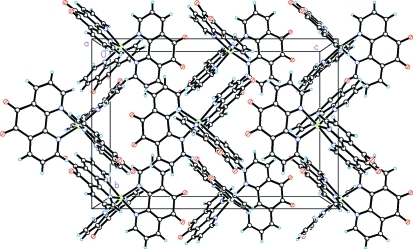
The packing diagram viewed along the a axis.

## supplementary crystallographic information

### Comment

The coordination chemistry of the chelating ligand 1,10-phenanthroline-5,6-dione has been examined in several studies (Khalaji *et al.*, 2007; Ma *et al.*, 2010; Rezvani *et al.*, 2010). Carrying an *O*-quinone and *N*-pyridine moiety to form stable complexes with a wide variety of metal ions, this ligand is considered to have many interesting biological and electrochemical properties (Boghaei & Asl, 2007; Goss & Abruna, 1985; Kou *et al.*, 2009). As far as we know, 1,10-phenanthroline-5,6-dione usually binds to metals through its imine N atoms, in some cases both the N and O donors are used simultaneously (Brechin *et al.*, 2008). In this paper, we report the crystal structure of the title compound in the hope of exploring some spectroscopic properties of d^10^ metal complexes.

The title compound consists of a [Zn(C_12_H_6_N_2_O_2_)_3_]^2+^ complex cations, two perchlorate anions and an acetonitrile solvent molecule. As shown in Fig. 1, three bidentate 1,10-phenanthroline-5,6-dione ligands are coordinated to the Zn^II^ atom solely *via* two N atoms. The coordination geometry around the Zn^II^ atom is distorted octahedral, with bite angles of 76.88 (18)–77.9 (2)° for the three bidentate ligands. The *cis* bond angles at the Zn^II^ atom fall in the range of 76.85 (18)–100.0 (2)° and the *trans* bond angles are 164.96 (17), 170.57 (18) and 173.9 (2)°, suggesting a significant deviation from a perfect octahedral coordination. The Zn—N bond lengths range from 2.117 (5) to 2.202 (5) Å, with an average of 2.158 (5) Å. There is no strong hydrogen bond in the crystal, as shown in Fig. 2. Weak intermolecular C—H···O hydrogen bonds (Table 1) and O···π and N···π interactions [O···centroid distances = 2.907 (5) and 2.843 (7) Å, N···centroid distance = 2.861 (10) Å] link the complex molecules, perchlorate anions and acetonitrile solvent molecules into a three-dimensional network.

### Experimental

The title compound was prepared by adding a solution of Zn(ClO_4_)_2_.2H_2_O (28.2 mg, 0.1 mmol) in 3 ml acetonitrile to a solution containing 1,10-phenanthroline-5,6-dione (63.1 mg, 0.3 mmol) in 12 ml acetonitrile. The mixture was stirred at room temperature for 5 h and then filtered. Yellow crystals suitable for X-ray diffraction were obtained by slow evaporation of the solvent after two days. They were collected by filtration, washed with diethyl ether and dried in air.

### Refinement

H atoms were positioned geometrically and refined as riding atoms, with C—H = 0.93 (aromatic) and 0.96 (methyl) Å and with *U*_iso_(H) = 1.2(1.5 for methyl)*U*_eq_(C). The highest residual electron density was found at 2.62 Å from H32 atom and the deepest hole at 0.36 Å from Cl1 atom.

### Crystal data

**Table:**

[Zn(C_12_H_6_N_2_O_2_)_3_](ClO_4_)_2_·C_2_H_3_N	*F*(000) = 1896
*M**_r_* = 935.91	*D*_x_ = 1.598 Mg m^−^^3^
Orthorhombic, *P*2_1_2_1_2_1_	Mo *K*α radiation, λ = 0.71073 Å
Hall symbol: P 2ac 2ab	Cell parameters from 5261 reflections
*a* = 13.446 (6) Å	θ = 2.3–22.5°
*b* = 14.125 (6) Å	µ = 0.85 mm^−^^1^
*c* = 20.483 (8) Å	*T* = 296 K
*V* = 3890 (3) Å^3^	Prism, yellow
*Z* = 4	0.30 × 0.12 × 0.12 mm

### Data collection

**Table:**

Bruker APEXII CCD diffractometer	9569 independent reflections
Radiation source: fine-focus sealed tube	5533 reflections with *I* > 2σ(*I*)
graphite	*R*_int_ = 0.079
φ and ω scans	θ_max_ = 28.6°, θ_min_ = 1.8°
Absorption correction: multi-scan (*SADABS*; Sheldrick, 1996)	*h* = −8→17
*T*_min_ = 0.785, *T*_max_ = 0.905	*k* = −18→18
23200 measured reflections	*l* = −27→24

### Refinement

**Table:**

Refinement on *F*^2^	Hydrogen site location: inferred from neighbouring sites
Least-squares matrix: full	H-atom parameters constrained
*R*[*F*^2^ > 2σ(*F*^2^)] = 0.075	*w* = 1/[σ^2^(*F*_o_^2^) + (0.1231*P*)^2^] where *P* = (*F*_o_^2^ + 2*F*_c_^2^)/3
*wR*(*F*^2^) = 0.220	(Δ/σ)_max_ = 0.001
*S* = 0.97	Δρ_max_ = 1.16 e Å^−^^3^
9569 reflections	Δρ_min_ = −0.39 e Å^−^^3^
562 parameters	Extinction correction: *SHELXL97* (Sheldrick, 2008), Fc^*^=kFc[1+0.001xFc^2^λ^3^/sin(2θ)]^-1/4^
1 restraint	Extinction coefficient: 0.0031 (7)
Primary atom site location: structure-invariant direct methods	Absolute structure: Flack (1983), 4095 Friedel pairs
Secondary atom site location: difference Fourier map	Flack parameter: 0.12 (2)

### Fractional atomic coordinates and isotropic or equivalent isotropic displacement parameters (Å^2^)

**Table:**

	*x*	*y*	*z*	*U*_iso_*/*U*_eq_	
Zn1	0.30685 (6)	0.98904 (5)	0.06979 (3)	0.0455 (2)	
Cl1	0.67594 (16)	0.99782 (14)	0.20658 (10)	0.0753 (6)	
Cl2	0.34979 (14)	0.98420 (14)	0.80652 (8)	0.0625 (5)	
C1	0.0895 (6)	0.9679 (5)	0.1240 (3)	0.064 (2)	
H14	0.1171	0.9259	0.1540	0.077*	
C2	−0.0107 (6)	0.9882 (7)	0.1292 (4)	0.072 (2)	
H15	−0.0495	0.9619	0.1621	0.086*	
C3	−0.0496 (7)	1.0470 (6)	0.0851 (3)	0.068 (2)	
H34	−0.1173	1.0604	0.0864	0.082*	
C4	0.0077 (6)	1.0874 (5)	0.0385 (3)	0.0545 (16)	
C5	0.1094 (5)	1.0680 (4)	0.0373 (3)	0.0454 (15)	
C6	−0.0314 (6)	1.1548 (6)	−0.0103 (3)	0.0589 (18)	
C7	0.0375 (6)	1.2115 (5)	−0.0490 (4)	0.0607 (19)	
C8	0.1777 (5)	1.1146 (4)	−0.0073 (3)	0.0434 (14)	
C9	0.1438 (6)	1.1861 (4)	−0.0494 (3)	0.0500 (16)	
C10	0.2105 (7)	1.2283 (5)	−0.0905 (3)	0.061 (2)	
H12	0.1899	1.2756	−0.1191	0.073*	
C11	0.3100 (7)	1.1998 (5)	−0.0890 (3)	0.0560 (16)	
H9	0.3567	1.2267	−0.1169	0.067*	
C12	0.3362 (6)	1.1323 (5)	−0.0463 (3)	0.0537 (17)	
H23	0.4026	1.1138	−0.0453	0.064*	
C13	0.2120 (8)	0.8282 (5)	−0.0163 (3)	0.073 (3)	
H32	0.1532	0.8421	0.0056	0.088*	
C14	0.2102 (9)	0.7601 (5)	−0.0669 (4)	0.082 (3)	
H21	0.1518	0.7281	−0.0773	0.098*	
C15	0.2969 (11)	0.7423 (5)	−0.1005 (4)	0.092 (4)	
H25	0.2984	0.6983	−0.1343	0.111*	
C16	0.3771 (8)	0.7886 (5)	−0.0837 (3)	0.069 (2)	
C17	0.3754 (7)	0.8544 (4)	−0.0322 (3)	0.055 (2)	
C18	0.4704 (11)	0.7718 (7)	−0.1200 (5)	0.098 (4)	
C19	0.5643 (12)	0.8214 (10)	−0.0980 (5)	0.121 (5)	
C20	0.4652 (6)	0.9057 (5)	−0.0147 (3)	0.0535 (17)	
C21	0.5537 (8)	0.8898 (6)	−0.0445 (4)	0.078 (2)	
C22	0.6343 (8)	0.9412 (9)	−0.0293 (5)	0.104 (3)	
H33	0.6935	0.9344	−0.0523	0.124*	
C23	0.6262 (7)	1.0052 (8)	0.0220 (5)	0.094 (3)	
H36	0.6809	1.0397	0.0363	0.113*	
C24	0.5360 (6)	1.0154 (6)	0.0506 (4)	0.0677 (19)	
H26	0.5304	1.0583	0.0849	0.081*	
C25	0.3351 (6)	0.8160 (5)	0.1594 (3)	0.0554 (18)	
H17	0.3106	0.7802	0.1249	0.067*	
C26	0.3698 (7)	0.7699 (5)	0.2160 (4)	0.067 (2)	
H27	0.3689	0.7042	0.2182	0.080*	
C27	0.4033 (6)	0.8194 (5)	0.2656 (3)	0.0543 (17)	
H20	0.4261	0.7888	0.3029	0.065*	
C28	0.4046 (5)	0.9162 (5)	0.2621 (3)	0.0463 (15)	
C29	0.3698 (5)	0.9579 (4)	0.2062 (3)	0.0421 (14)	
C30	0.4395 (6)	0.9750 (5)	0.3177 (3)	0.0555 (17)	
C31	0.4307 (6)	1.0827 (6)	0.3127 (3)	0.0597 (19)	
C32	0.3645 (5)	1.0629 (4)	0.1986 (3)	0.0410 (13)	
C33	0.3887 (5)	1.1216 (5)	0.2506 (3)	0.0484 (15)	
C34	0.3719 (7)	1.2171 (5)	0.2433 (3)	0.062 (2)	
H7	0.3864	1.2586	0.2772	0.075*	
C35	0.3347 (6)	1.2500 (5)	0.1872 (3)	0.061 (2)	
H24	0.3202	1.3140	0.1823	0.073*	
C36	0.3182 (6)	1.1875 (4)	0.1369 (3)	0.0540 (17)	
H8	0.2952	1.2108	0.0972	0.065*	
C37	0.5814 (10)	0.1897 (9)	0.8669 (5)	0.113 (4)	
C38	0.5824 (12)	0.1612 (15)	0.8005 (6)	0.193 (9)	
H38A	0.6089	0.2114	0.7741	0.289*	
H38B	0.5159	0.1470	0.7866	0.289*	
H38C	0.6233	0.1058	0.7958	0.289*	
N1	0.1489 (4)	1.0048 (4)	0.0788 (2)	0.0461 (11)	
N2	0.2743 (4)	1.0902 (3)	−0.0054 (2)	0.0449 (12)	
N3	0.2912 (6)	0.8710 (4)	0.0003 (2)	0.0595 (17)	
N4	0.4580 (5)	0.9700 (4)	0.0335 (2)	0.0522 (14)	
N5	0.3368 (5)	0.9083 (3)	0.1546 (2)	0.0467 (14)	
N6	0.3339 (4)	1.0955 (3)	0.1430 (2)	0.0436 (13)	
N7	0.5798 (8)	0.2050 (7)	0.9193 (4)	0.105 (3)	
O1	−0.1211 (5)	1.1624 (5)	−0.0184 (3)	0.0876 (18)	
O2	0.0074 (5)	1.2760 (4)	−0.0809 (3)	0.0803 (17)	
O3	0.4753 (8)	0.7195 (6)	−0.1651 (4)	0.140 (4)	
O4	0.6416 (9)	0.7953 (10)	−0.1217 (5)	0.203 (6)	
O5	0.4748 (5)	0.9415 (4)	0.3661 (2)	0.0744 (16)	
O6	0.4577 (5)	1.1315 (4)	0.3557 (2)	0.0781 (17)	
O7	0.652 (2)	1.0724 (6)	0.1856 (7)	0.351 (17)	
O8	0.7584 (9)	0.966 (2)	0.1956 (13)	0.42 (2)	
O9	0.6192 (12)	0.9199 (6)	0.1767 (7)	0.200 (6)	
O10	0.6744 (18)	0.9940 (10)	0.2718 (6)	0.312 (12)	
O11	0.3492 (6)	0.9109 (5)	0.7595 (3)	0.105 (2)	
O12	0.2624 (5)	0.9759 (4)	0.8471 (3)	0.0815 (16)	
O13	0.3483 (6)	1.0713 (4)	0.7743 (3)	0.094 (2)	
O14	0.4366 (5)	0.9798 (5)	0.8447 (3)	0.096 (2)	

### Atomic displacement parameters (Å^2^)

**Table:**

	*U*^11^	*U*^22^	*U*^33^	*U*^12^	*U*^13^	*U*^23^
Zn1	0.0575 (4)	0.0389 (3)	0.0400 (3)	0.0035 (3)	−0.0038 (3)	0.0016 (3)
Cl1	0.0823 (14)	0.0599 (11)	0.0837 (11)	0.0149 (10)	0.0214 (10)	0.0215 (9)
Cl2	0.0698 (11)	0.0661 (11)	0.0515 (7)	−0.0053 (10)	−0.0100 (8)	0.0028 (8)
C1	0.071 (5)	0.074 (5)	0.049 (3)	−0.011 (4)	0.004 (4)	0.020 (3)
C2	0.068 (5)	0.085 (5)	0.062 (4)	0.000 (5)	0.015 (4)	0.010 (4)
C3	0.061 (5)	0.089 (5)	0.054 (4)	0.008 (4)	0.007 (4)	−0.004 (3)
C4	0.059 (4)	0.060 (4)	0.045 (3)	0.001 (3)	−0.005 (3)	−0.004 (3)
C5	0.061 (4)	0.041 (3)	0.034 (3)	−0.005 (3)	−0.005 (3)	−0.001 (2)
C6	0.056 (5)	0.071 (5)	0.049 (3)	0.018 (4)	0.003 (3)	0.000 (3)
C7	0.070 (5)	0.045 (4)	0.067 (4)	0.002 (4)	−0.013 (4)	−0.004 (3)
C8	0.057 (4)	0.036 (3)	0.038 (3)	0.002 (3)	−0.003 (3)	−0.003 (2)
C9	0.065 (5)	0.039 (3)	0.045 (3)	0.003 (3)	−0.010 (3)	−0.002 (2)
C10	0.098 (7)	0.046 (3)	0.039 (3)	0.000 (4)	0.002 (4)	0.012 (2)
C11	0.066 (5)	0.050 (4)	0.052 (3)	−0.008 (4)	−0.001 (4)	0.010 (2)
C12	0.061 (5)	0.058 (4)	0.043 (3)	−0.002 (3)	−0.007 (3)	−0.001 (3)
C13	0.111 (8)	0.056 (4)	0.052 (4)	−0.019 (5)	0.003 (5)	−0.002 (3)
C14	0.119 (9)	0.057 (4)	0.069 (5)	−0.015 (5)	−0.032 (6)	−0.001 (4)
C15	0.171 (12)	0.045 (4)	0.061 (4)	−0.022 (6)	−0.003 (7)	−0.006 (3)
C16	0.108 (7)	0.046 (4)	0.054 (4)	0.014 (4)	−0.013 (4)	−0.005 (3)
C17	0.094 (6)	0.035 (3)	0.037 (3)	0.016 (3)	−0.007 (4)	0.000 (2)
C18	0.145 (11)	0.069 (6)	0.081 (6)	0.035 (7)	0.003 (7)	−0.002 (5)
C19	0.141 (12)	0.154 (11)	0.068 (5)	0.079 (10)	0.003 (7)	−0.022 (6)
C20	0.071 (5)	0.044 (3)	0.045 (3)	0.022 (3)	−0.002 (3)	0.006 (3)
C21	0.087 (6)	0.072 (5)	0.075 (5)	0.039 (5)	−0.003 (5)	−0.006 (4)
C22	0.067 (6)	0.148 (10)	0.097 (7)	0.048 (6)	0.002 (6)	0.020 (7)
C23	0.063 (5)	0.112 (8)	0.108 (7)	0.002 (6)	0.001 (5)	0.008 (7)
C24	0.071 (5)	0.060 (4)	0.072 (4)	0.001 (4)	−0.017 (4)	−0.005 (3)
C25	0.068 (5)	0.044 (4)	0.054 (3)	0.004 (3)	0.001 (3)	0.008 (3)
C26	0.093 (6)	0.045 (4)	0.062 (4)	0.019 (4)	0.014 (4)	0.018 (3)
C27	0.062 (5)	0.059 (4)	0.042 (3)	0.010 (3)	0.001 (3)	0.012 (3)
C28	0.050 (4)	0.053 (4)	0.036 (3)	0.005 (3)	0.006 (3)	0.004 (2)
C29	0.048 (4)	0.040 (3)	0.039 (3)	0.001 (2)	0.006 (3)	0.004 (2)
C30	0.059 (4)	0.067 (5)	0.041 (3)	0.000 (4)	0.003 (3)	0.010 (3)
C31	0.060 (5)	0.077 (5)	0.041 (3)	−0.015 (4)	−0.002 (3)	−0.001 (3)
C32	0.046 (4)	0.035 (3)	0.042 (3)	0.002 (2)	0.005 (3)	−0.002 (2)
C33	0.051 (4)	0.050 (4)	0.045 (3)	−0.004 (3)	0.003 (3)	−0.010 (3)
C34	0.087 (6)	0.049 (4)	0.051 (4)	−0.012 (4)	0.008 (4)	−0.012 (3)
C35	0.077 (6)	0.040 (3)	0.066 (4)	0.001 (3)	0.008 (4)	−0.005 (3)
C36	0.075 (5)	0.041 (3)	0.046 (3)	0.003 (3)	0.004 (4)	0.003 (2)
C37	0.123 (10)	0.134 (10)	0.083 (6)	−0.054 (8)	0.016 (7)	−0.012 (6)
C38	0.132 (12)	0.35 (3)	0.098 (8)	−0.110 (16)	0.031 (9)	−0.062 (12)
N1	0.047 (3)	0.045 (3)	0.046 (2)	−0.005 (2)	−0.006 (2)	0.011 (2)
N2	0.058 (4)	0.044 (3)	0.034 (2)	0.003 (2)	0.002 (2)	0.0067 (19)
N3	0.096 (5)	0.040 (3)	0.042 (3)	−0.011 (3)	−0.012 (3)	−0.001 (2)
N4	0.067 (4)	0.047 (3)	0.043 (2)	0.001 (3)	−0.001 (3)	−0.006 (2)
N5	0.067 (4)	0.032 (2)	0.041 (2)	0.006 (2)	−0.001 (3)	0.0039 (18)
N6	0.056 (4)	0.035 (2)	0.039 (2)	0.002 (2)	0.007 (2)	0.0011 (18)
N7	0.114 (7)	0.123 (7)	0.079 (5)	−0.009 (6)	0.007 (6)	−0.028 (5)
O1	0.072 (4)	0.116 (5)	0.075 (3)	0.020 (4)	0.001 (3)	0.012 (3)
O2	0.081 (4)	0.061 (3)	0.098 (4)	0.017 (3)	−0.024 (4)	0.011 (3)
O3	0.208 (11)	0.110 (6)	0.102 (5)	0.059 (6)	0.006 (6)	−0.050 (4)
O4	0.146 (9)	0.318 (15)	0.146 (8)	0.132 (10)	−0.013 (7)	−0.111 (9)
O5	0.084 (4)	0.091 (4)	0.049 (2)	−0.005 (3)	−0.012 (3)	0.017 (2)
O6	0.094 (5)	0.091 (4)	0.049 (2)	−0.007 (4)	−0.013 (3)	−0.011 (3)
O7	0.70 (5)	0.064 (6)	0.291 (15)	0.024 (12)	−0.32 (2)	−0.017 (7)
O8	0.087 (8)	0.65 (5)	0.54 (4)	0.020 (16)	0.048 (14)	−0.36 (4)
O9	0.290 (17)	0.062 (5)	0.249 (12)	−0.044 (7)	0.061 (12)	−0.011 (6)
O10	0.59 (4)	0.191 (12)	0.152 (9)	0.00 (2)	0.128 (16)	0.040 (9)
O11	0.101 (5)	0.098 (5)	0.115 (5)	−0.006 (4)	−0.002 (5)	−0.047 (4)
O12	0.098 (4)	0.085 (4)	0.061 (3)	−0.004 (3)	0.003 (3)	0.006 (3)
O13	0.128 (6)	0.084 (4)	0.070 (3)	0.009 (4)	−0.007 (4)	0.031 (3)
O14	0.081 (4)	0.119 (5)	0.088 (4)	−0.012 (4)	−0.030 (3)	0.034 (4)

### Geometric parameters (Å, °)

**Table:**

Zn1—N5	2.117 (5)	C16—C18	1.478 (15)
Zn1—N1	2.143 (5)	C17—N3	1.334 (10)
Zn1—N2	2.146 (5)	C17—C20	1.453 (11)
Zn1—N6	2.154 (5)	C18—O3	1.184 (11)
Zn1—N4	2.181 (6)	C18—C19	1.514 (19)
Zn1—N3	2.202 (5)	C19—O4	1.204 (15)
Cl1—O7	1.180 (10)	C19—C21	1.468 (13)
Cl1—O8	1.219 (14)	C20—N4	1.345 (8)
Cl1—O10	1.338 (12)	C20—C21	1.357 (12)
Cl1—O9	1.472 (12)	C21—C22	1.341 (16)
Cl2—O13	1.396 (6)	C22—C23	1.391 (15)
Cl2—O14	1.407 (6)	C22—H33	0.9300
Cl2—O11	1.415 (6)	C23—C24	1.355 (12)
Cl2—O12	1.444 (6)	C23—H36	0.9300
C1—N1	1.330 (8)	C24—N4	1.277 (9)
C1—C2	1.382 (12)	C24—H26	0.9300
C1—H14	0.9300	C25—N5	1.307 (8)
C2—C3	1.334 (11)	C25—C26	1.409 (9)
C2—H15	0.9300	C25—H17	0.9300
C3—C4	1.353 (10)	C26—C27	1.312 (11)
C3—H34	0.9300	C26—H27	0.9300
C4—C5	1.395 (10)	C27—C28	1.369 (9)
C4—C6	1.476 (10)	C27—H20	0.9300
C5—N1	1.342 (7)	C28—C29	1.370 (8)
C5—C8	1.453 (9)	C28—C30	1.485 (9)
C6—O1	1.221 (10)	C29—N5	1.343 (8)
C6—C7	1.460 (11)	C29—C32	1.492 (8)
C7—O2	1.191 (8)	C30—O5	1.197 (8)
C7—C9	1.473 (11)	C30—C31	1.529 (11)
C8—N2	1.345 (9)	C31—O6	1.176 (8)
C8—C9	1.404 (8)	C31—C33	1.495 (10)
C9—C10	1.366 (10)	C32—N6	1.296 (7)
C10—C11	1.398 (13)	C32—C33	1.389 (8)
C10—H12	0.9300	C33—C34	1.376 (10)
C11—C12	1.342 (9)	C34—C35	1.336 (10)
C11—H9	0.9300	C34—H7	0.9300
C12—N2	1.321 (8)	C35—C36	1.375 (9)
C12—H23	0.9300	C35—H24	0.9300
C13—N3	1.271 (11)	C36—N6	1.323 (8)
C13—C14	1.414 (11)	C36—H8	0.9300
C13—H32	0.9300	C37—N7	1.096 (12)
C14—C15	1.377 (16)	C37—C38	1.419 (15)
C14—H21	0.9300	C38—H38A	0.9600
C15—C16	1.307 (14)	C38—H38B	0.9600
C15—H25	0.9300	C38—H38C	0.9600
C16—C17	1.406 (9)		
			
N5—Zn1—N1	100.0 (2)	C16—C18—C19	118.9 (8)
N5—Zn1—N2	170.57 (18)	O4—C19—C21	125.8 (15)
N1—Zn1—N2	77.9 (2)	O4—C19—C18	117.3 (12)
N5—Zn1—N6	76.85 (18)	C21—C19—C18	116.5 (11)
N1—Zn1—N6	92.0 (2)	N4—C20—C21	120.3 (8)
N2—Zn1—N6	93.96 (18)	N4—C20—C17	117.3 (6)
N5—Zn1—N4	92.1 (2)	C21—C20—C17	122.4 (7)
N1—Zn1—N4	164.96 (17)	C22—C21—C20	121.0 (9)
N2—Zn1—N4	91.6 (2)	C22—C21—C19	116.8 (11)
N6—Zn1—N4	99.5 (2)	C20—C21—C19	122.0 (11)
N5—Zn1—N3	98.09 (19)	C21—C22—C23	117.6 (10)
N1—Zn1—N3	92.3 (2)	C21—C22—H33	121.2
N2—Zn1—N3	91.20 (19)	C23—C22—H33	121.2
N6—Zn1—N3	173.9 (2)	C24—C23—C22	117.8 (10)
N4—Zn1—N3	77.0 (2)	C24—C23—H36	121.1
O7—Cl1—O8	120 (2)	C22—C23—H36	121.1
O7—Cl1—O10	113.3 (10)	N4—C24—C23	124.3 (8)
O8—Cl1—O10	100.6 (16)	N4—C24—H26	117.9
O7—Cl1—O9	112.2 (9)	C23—C24—H26	117.9
O8—Cl1—O9	96.8 (12)	N5—C25—C26	121.1 (7)
O10—Cl1—O9	112.1 (10)	N5—C25—H17	119.4
O13—Cl2—O14	108.3 (5)	C26—C25—H17	119.4
O13—Cl2—O11	108.8 (4)	C27—C26—C25	120.3 (7)
O14—Cl2—O11	110.6 (5)	C27—C26—H27	119.8
O13—Cl2—O12	109.4 (4)	C25—C26—H27	119.8
O14—Cl2—O12	110.6 (3)	C26—C27—C28	119.7 (6)
O11—Cl2—O12	109.1 (4)	C26—C27—H20	120.1
N1—C1—C2	123.9 (7)	C28—C27—H20	120.1
N1—C1—H14	118.1	C27—C28—C29	118.0 (6)
C2—C1—H14	118.1	C27—C28—C30	121.5 (6)
C3—C2—C1	117.4 (7)	C29—C28—C30	120.5 (6)
C3—C2—H15	121.3	N5—C29—C28	123.0 (5)
C1—C2—H15	121.3	N5—C29—C32	114.9 (5)
C2—C3—C4	121.1 (8)	C28—C29—C32	122.1 (6)
C2—C3—H34	119.4	O5—C30—C28	122.6 (7)
C4—C3—H34	119.4	O5—C30—C31	118.7 (7)
C3—C4—C5	119.2 (7)	C28—C30—C31	118.7 (6)
C3—C4—C6	123.1 (7)	O6—C31—C33	122.5 (7)
C5—C4—C6	117.6 (6)	O6—C31—C30	120.6 (7)
N1—C5—C4	120.5 (6)	C33—C31—C30	116.9 (6)
N1—C5—C8	116.7 (6)	N6—C32—C33	122.5 (5)
C4—C5—C8	122.8 (6)	N6—C32—C29	117.4 (5)
O1—C6—C7	120.3 (7)	C33—C32—C29	120.1 (6)
O1—C6—C4	120.1 (8)	C34—C33—C32	117.6 (6)
C7—C6—C4	119.7 (7)	C34—C33—C31	121.0 (6)
O2—C7—C6	120.1 (8)	C32—C33—C31	121.4 (6)
O2—C7—C9	120.8 (8)	C35—C34—C33	119.8 (6)
C6—C7—C9	119.1 (6)	C35—C34—H7	120.1
N2—C8—C9	121.1 (6)	C33—C34—H7	120.1
N2—C8—C5	118.4 (5)	C34—C35—C36	118.7 (6)
C9—C8—C5	120.5 (6)	C34—C35—H24	120.6
C10—C9—C8	118.6 (7)	C36—C35—H24	120.6
C10—C9—C7	122.3 (6)	N6—C36—C35	122.3 (6)
C8—C9—C7	119.1 (6)	N6—C36—H8	118.8
C9—C10—C11	119.3 (6)	C35—C36—H8	118.8
C9—C10—H12	120.3	N7—C37—C38	174.8 (16)
C11—C10—H12	120.3	C37—C38—H38A	109.5
C12—C11—C10	118.1 (7)	C37—C38—H38B	109.5
C12—C11—H9	121.0	H38A—C38—H38B	109.5
C10—C11—H9	121.0	C37—C38—H38C	109.5
N2—C12—C11	124.6 (7)	H38A—C38—H38C	109.5
N2—C12—H23	117.7	H38B—C38—H38C	109.5
C11—C12—H23	117.7	C1—N1—C5	117.7 (6)
N3—C13—C14	122.3 (10)	C1—N1—Zn1	127.9 (5)
N3—C13—H32	118.9	C5—N1—Zn1	114.0 (4)
C14—C13—H32	118.9	C12—N2—C8	118.4 (5)
C15—C14—C13	118.4 (9)	C12—N2—Zn1	128.7 (5)
C15—C14—H21	120.8	C8—N2—Zn1	112.8 (4)
C13—C14—H21	120.8	C13—N3—C17	119.6 (6)
C16—C15—C14	118.4 (8)	C13—N3—Zn1	127.8 (6)
C16—C15—H25	120.8	C17—N3—Zn1	112.0 (5)
C14—C15—H25	120.8	C24—N4—C20	118.8 (7)
C15—C16—C17	121.0 (10)	C24—N4—Zn1	127.6 (5)
C15—C16—C18	119.1 (9)	C20—N4—Zn1	113.5 (5)
C17—C16—C18	119.9 (9)	C25—N5—C29	117.9 (5)
N3—C17—C16	120.3 (8)	C25—N5—Zn1	126.5 (4)
N3—C17—C20	119.6 (6)	C29—N5—Zn1	115.3 (4)
C16—C17—C20	120.1 (8)	C32—N6—C36	118.9 (5)
O3—C18—C16	122.7 (13)	C32—N6—Zn1	114.7 (4)
O3—C18—C19	118.4 (12)	C36—N6—Zn1	126.4 (4)

### Hydrogen-bond geometry (Å, °)

**Table:**

*D*—H···*A*	*D*—H	H···*A*	*D*···*A*	*D*—H···*A*
C1—H14···O13^i^	0.93	2.51	3.238 (9)	135
C2—H15···O3^ii^	0.93	2.58	3.030 (13)	110
C10—H12···O7^iii^	0.93	2.59	3.513 (14)	170
C25—H17···O4^ii^	0.93	2.51	3.136 (15)	125
C24—H26···O2^iv^	0.93	2.36	3.036 (10)	129
C34—H7···O9^v^	0.93	2.47	3.302 (12)	150
C38—H38A···O3^vi^	0.96	2.51	2.995 (16)	112
C38—H38B···O13^vii^	0.96	2.51	3.436 (19)	163

Symmetry codes: (i) −*x*+1/2, −*y*+2, *z*−1/2; (ii) *x*−1/2, −*y*+3/2, −*z*; (iii) *x*−1/2, −*y*+5/2, −*z*; (iv) *x*+1/2, −*y*+5/2, −*z*; (v) −*x*+1, *y*+1/2, −*z*+1/2; (vi) −*x*+1, *y*−1/2, −*z*+1/2; (vii) *x*, *y*−1, *z*.
